# Family Functioning and Optimism as Protective Factors of Life Satisfaction Among Stroke Patients During the COVID-19 Epidemic in Shenyang, China

**DOI:** 10.3389/fpubh.2022.738634

**Published:** 2022-04-26

**Authors:** Yuequn Song, Can Cui, Yajing Jia, Weiyu Zhang, Lifang Meng, Kristin K. Sznajder, Yuanyuan Xu, Xiaoshi Yang

**Affiliations:** ^1^Department of Neurosurgery, The Fourth Affiliated Hospital of China Medical University, Shenyang, China; ^2^Department of Social Medicine, College of Health Management, China Medical University, Shenyang, China; ^3^Department of Scientific Research Management, China Medical University, Shenyang, China; ^4^Department of Public Health Sciences, College of Medicine, Pennsylvania State University, Hershey, PA, United States; ^5^Group of Chronic Disease and Environmental Genomics, China Medical University School of Public Health, Shenyang, China

**Keywords:** COVID-19, life satisfaction, family functioning, optimism, stroke patients

## Abstract

The coronavirus disease-19 (COVID-19) pandemic may result in detrimental consequences for stroke patient's wellbeing. Family functioning and optimism could help stroke patients cope with crises leading to possible improvements in life satisfaction. This study aims to explore the protective effects of family functioning and optimism on life satisfaction among stroke patients during the COVID-19 pandemic in China. This study was designed as a cross-sectional survey. A total of 207 stroke inpatients who were receiving pharmacotherapy and rehabilitation in general public hospital of Liaoning province during the COVID-19 pandemic in China were consecutive selected and interviewed by online questionnaires via the WeChat platform effectively from April 8 to 30, 2020. The scales included: Satisfaction with Life Scale (SWLS), Family Adaptation, Partnership, Growth, Affection, and Resolve (APGAR) Scale and Revised Life Orientation Test (LOT-R). Hierarchical multiple regression (HMR) analysis was conducted to test the associated factors of life satisfaction. Stroke patient's life satisfaction was at a high level (Mean = 26.46, SD = 6.23) during the pandemic. Stroke patient's residence, duration of stroke, stroke type, and community shut down measures were the strong predictors of life satisfaction. Family functioning and optimism increased life satisfaction among stroke patients. This study contributes to the research on the association between family functioning and optimism on life satisfaction among stroke patients during the COVID-19 pandemic. Interventions that improve family functioning and enhance optimism should be provided in order to elevate life satisfaction for stroke patients.

## Introduction

With the surge of people infected with severe acute respiratory syndrome coronavirus 2 (SARS-CoV-2) the virus that causes coronavirus disease 2019 (COVID-19) and the stringent implementation of public health restrictions (e.g., traffic restrictions, home quarantine, and physical distancing legislation) have broadened the impact of the pandemic to a point where it will affect life satisfaction for all members of society, especially for patients with cerebrovascular disease (1–4). Stroke as the most common cerebrovascular disease in adults and is highly correlated with physical disability that may require long-term treatment and rehabilitation, which may severely affect the life satisfaction of patients (5).

According to China Stroke Statistics 2019 by Wang, nearly 110,000 patients suffer from strokes every year, accounting for a total of 30,000 deaths and 500,000 stroke related disabilities annually (6). The experience of a stroke brings a heavy burden financially, mentally, and physically to detrimentally affected patients and their family members (7). Life satisfaction is the multidimensional measurement of subjective wellbeing. In order to measure life satisfaction, one must reflect on their overall health, interpersonal relationships, socioeconomic status, ability in self-care, and leisure activities (8–10). Thus, life satisfaction was used as a measure to investigate to what extent the epidemic response, family functioning, and optimism have impacted stroke patients during the COVID-19 epidemic.

The COVID-19 pandemic has brought about new social environments characterized by curfews and physical distancing, which could affect health care services and treatment procedures, resulting in the reduction of life satisfaction (11). The uncertainty and anxiety surrounding the COVID-19 pandemic has caused psychological distress for stroke patients who are under clinical treatment in hospitals, which may negatively affect their subjective health, life satisfaction, and overall wellbeing (12). Data from Vestling et al. (13) showed that, compared to stroke patients without distress or fear regarding the COVID-19 pandemic, patients with moderate or high levels of distress or fear could have a lower level of global satisfaction, which could be particularly problematic among stroke patients. Stroke patients with low access to health care may be especially vulnerable during the COVID-19 pandemic and have an increased risk for poor subjective wellbeing and psychological health (14). In particular, the COVID-19 pandemic has the potential to produce a vast array of psychological health challenges such as adjustment disorder, fear, and anxiety, which may negatively impact the life satisfaction of stroke patients (15, 16). Further, stroke patients who are suffering from greater impairments, including extensive reductions in daily activities, have poorer life satisfaction, compared with the general population under the COVID-19 pandemic (17). Studies have found that the clinical visit rate of stroke disease decreased sharply during the COVID-19 pandemic, which could lead to reduced life satisfaction for chronic patients (18).

In China, caring for stroke patients is expected to be done by family members who are considered the major source of financial, material and psychological support (19). A great number of studies have examined the effect of family functioning on stroke patient's life satisfaction. Olson Circumplex model is a theoretical framework that pays attention to interpret family balance relationship and individual wellbeing, according to the Olson Circumplex model, balanced family characterized without too little interaction or too much consensus within the family can positively influence individual's wellbeing and life satisfaction (20). Family functioning is defined by the relationships and roles within a family that contribute to problem management, adjustment to new family practices, and effective communication (21, 22). A study conducted by Koutra et al. (23) explored the role of family support in patients with chronic diseases, which revealed that patients with adequate supportive families tend to report lower emotional distress, which could improve their subjective wellbeing.

Based on the Broaden-and-Build Theory, positive emotions promote the individual's adaptation to the society by establishing lasting personal resources, such as social and psychological resources, which could finally predict their judgments of subjective wellbeing (24). Positive psychological resources may help stroke patients adapt to changing demands and improve emotional stability when confronted with mental disorders, which could enhance life satisfaction (25). Optimism, a favorable personality trait and positive psychological resource in which individuals generally hold the expectation of positive rather than the negative outcomes, together with other positive psychological responses to the COVID-19 pandemic have been found to improve subjective wellbeing and mental health (26, 27).

Associated factors related to the COVID-19 pandemic including community shut-down measures, impact on individual's daily lives, risk of infection, and anxiety can be considered chronic stressors. Whereas, family functioning and optimism may be protective against negative emotions and improve life satisfaction (28, 29). However, to date, no studies have assessed life satisfaction and its associated factors including family functioning and optimism among Chinese stroke patients during the COVID-19 pandemic. To better support stroke patients during the COVID-19 pandemic in China, more information is needed on the factors associated with life satisfaction. We hypothesize that:

Demographic and clinical characteristics (including residence, duration of stroke, and stroke type) and epidemic responses (including community shut down measures) could affect life satisfaction;

Family functioning and optimism are positively associated with life satisfaction.

## Methods

### Participants and Procedure

A cross-sectional study was employed from April 8 to April 30, 2020, in the general public hospital of Liaoning, China. A total of 258 stroke inpatients who were receiving pharmacotherapy, rehabilitation and met the inclusion criteria were consecutive selected in this study and these participants were interviewed face-to-face by the trained investigators using a mobile phone questionnaire via the WeChat platform. The inclusion criteria of stroke patients were the following: age more than 20 years old; fluent in oral or written Chinese, and able to consent to join the study. The exclusion criteria were having a history of serious mental illness or a serious chronic illness including schizophrenia, bipolar disorder, Alzheimer's disease, hysteria, cancer, dementia, or severe hearing or vision impairment. The participant was informed of the research aims and that the questionnaire was anonymous prior to informed consent. The questionnaire took approximately 25 min to complete. The questionnaire had been preset for submission only after all the questions were answered within the range of the selected choices. Answers were not available if the questions were not completed. Therefore, the collected questionnaire had been filtered with data cleaning, checking the consistency and logicality of the answers, adjusting invalid and missing values.

### Instruments

The information collected on stroke patient's demographic and clinical characteristics included age, gender, marital status, education level, residence, monthly income, duration of stroke, stroke type, and activities of daily living (ADL). “Marital status” was grouped as “married” or “other.” “Residence” was classified as “urban” or “rural.” “Education level” was defined as “junior high school and below” or “senior high school and above.” “Monthly income (RMB)” was categorized as: “ ≤ 3,000 yuan,” “3,001~6,000 yuan” and “>6,000 yuan.” “Duration of stroke” was grouped as “ ≤ 2 weeks” or “more than 2 weeks.” “Stroke type” was classified as “hemorrhagic stroke” or “ischemic stroke.” “ADL scores” were categorized as “mild disability (ADL scores ≤ 26)” or “high disability (ADL scores > 26).”

The responses to the COVID-19 pandemic in stroke patients were measured by 4 questions: (1) Community shut-down measures (yes/no), (2) Daily life impact due to the pandemic (yes/no), (3) Perception about the risks of SARS-CoV-2 infection (yes/no), (4) Anxiety about the pandemic (yes/no).

The Family Adaptation, Partnership, Growth, Affection, and Resolve (FAPGAR) Scale was employed to assess the perception of family functioning (30). This scale included five items that were answered on a 3-point Likert scale, ranging from 0 (never) to 2 (always) (30 28). The Cronbach's alpha coefficient for this scale was 0.936.

Optimism was evaluated by the 6-item Revised Life Orientation Test (LOT-R) that assessed the generalized expectations for positive or negative outcomes. The LOT-R was rated on a 5-point Likert scale from 0 (strongly disagree) to 4 (strongly agree) and the total scores were summed after reverse coding three items (31). The total scores ranged from 0 to 24, with higher scores indicating a greater degree of optimism. The Cronbach's alpha coefficient for this scale was 0.810.

Life satisfaction was assessed by the Satisfaction with Life Scale (SWLS) which was developed by Diener and is widely applied as a valid and reliable measure of life satisfaction for a variety of populations (32). It comprised of 5 items, to which the participants gave their responses of agreement on a 7-point Likert scale from 1 (“strongly disagree”) to 7 (“strongly agree”) (33). The Cronbach's alpha for this scale was 0.972.

### Statistical Analysis

The statistical analyses were performed using SPSS 22.0 version statistical software for Windows. The radar chart was used to describe significant association factors of life satisfaction for stroke patients during the COVID-19 epidemic. *T*-tests and one-way ANOVA were conducted to compare differences in life satisfaction among the categorical groups. The Spearman correlation was employed to test the correlations between family functioning, optimism, and life satisfaction. Hierarchical multiple regression (HMR) analysis was performed to predict factors associated with life satisfaction, in which, life satisfaction was used as the dependent variable. And the independent variables were entered in four steps: Step 1: stroke patient's demographic and clinical variables; Step 2: epidemic responses; Step 3: family functioning; and Step 4: optimism. A two-tailed probability value of <0.05 was considered statistically significant.

## Results

### Description of the Stroke Patients and Life Satisfaction

**Figure 1:**
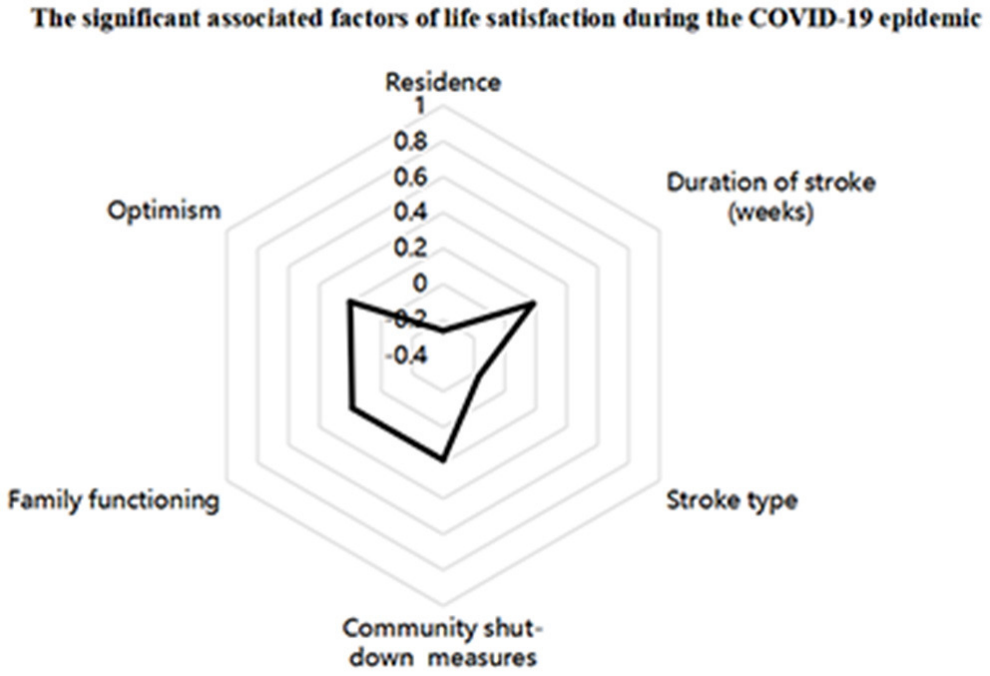
The radar chart of life satisfaction. Residence (Rural areas); Duration of stroke (>2 weeks); Stroke type (Ischemic stroke); Community shut-down measures (No).

Of the 258 stroke patients, 207 took part in this study and provided valid answers to the questionnaire, resulting in a valid response rate of 80.23%. The demographic and clinical characteristics, and epidemic responses of the stroke patients and the associations with life satisfaction are provided in Table 1. The mean age of patients was 64.7 years old (ranging from 33 to 93), and 63.3% of the patients were men. Approximately, 89.4% of the patients were currently married, and 93.7% had a monthly income of <6,000 yuan. Of the participants, 23.7% had a stroke duration of more than 2 weeks and 79.7% were diagnosed with a hemorrhagic stroke. Among the stroke patients, 79.2% experienced community shut-down measures during the research study. Patients whose duration of stroke was ≤ 2 weeks reported higher levels of life satisfaction than those whose stroke duration was more than 2 weeks (*P* < 0.05). Patients who lived in urban areas reported higher life satisfaction than those lived in rural areas (*P* < 0.05). Ischemic stroke patients exerted a lower level of life satisfaction than hemorrhagic stroke patients (*P* < 0.05). The stroke patients who lived in communities that were shut-down by the Chinese government had lower life satisfaction than their comparative group (*P* < 0.05). The radar chart of life satisfaction is shown in Figure 1.

**Table 1:** Characteristics of the stroke patients and distributions in life satisfaction (*N* = 207).

**Variables**	***N***	**%**	**Mean ±SD**
**Demographic and clinical characteristics**			
**Age (yr)**			
≤65	111	53.6	26.25 ± 6.78
>65	96	46.4	26.72 ± 5.57
**Gender**			
Male	131	63.3	26.08 ± 6.57
Female	76	36.7	27.13 ± 5.59
**Marital status**			
Married	185	89.4	26.29 ± 6.45
Others	22	10.6	27.95 ± 3.79
**Educational level**			
Junior high school and below	106	51.2	26.81 ± 6.19
Senior high school and above	101	48.8	26.10 ± 6.29
**Residence**			
Urban areas	195	94.2	26.87 ± 5.82^*^
Rural areas	12	5.8	19.83 ± 8.91
**Monthly income (RMB)**			
≤3,000	86	41.5	26.02 ± 6.32
3,001–6,000	108	52.2	26.70 ± 5.86
>6,000	13	6.3	27.46 ± 8.64
**Duration of stroke (weeks)**			
≤2weeks	158	76.3	25.98 ± 6.17
>2weeks	49	23.7	28.02 ± 6.25^*^
**Stroke type**			
Hemorrhagic stroke	165	79.7	26.98 ± 5.68^*^
Ischemic stroke	42	20.3	24.42 ± 7.80
**ADL scores**			
Mild disability (ADL scores ≤ 26)	117	56.5	26.31 ± 5.77
High disability (ADL scores > 26)	90	43.5	26.66 ± 6.82
**Epidemic responses**			
**Community shut-down measures**			
Yes	164	79.2	25.93 ± 6.47
No	43	20.8	28.51 ± 4.76^*^
**Felt daily life impacts of the pandemic**			
Yes	119	57.5	26.07 ± 6.04
No	88	42.5	27.00 ± 6.48
**Felt the risks of infection with the pandemic**			
Yes	155	74.9	26.73 ± 6.59
No	52	25.1	25.67 ± 5.00
**Felt being anxious about the epidemic**			
Yes	54	26.1	25.22 ± 6.77
No	153	73.9	26.90 ± 5.99

* *P < 0.05*.

### Correlations of Life Satisfaction and Continuous Variables

The Spearman correlation analyses of life satisfaction, family functioning, and optimism are presented in Table 2. Results revealed that family functioning (*r* = 0.305, *P* < 0.01) and optimism were both positively correlated with life satisfaction (*r* = 0.296, *P* < 0.01).

**Table 2:** The correlations of life satisfaction and continuous variables (*N* = 207).

**Variables**	**Mean**	**SD**	**Range**	**1**	**2**	**3**
1. Life satisfaction	26.46	6.23	5~35	1		
2. Family functioning	7.29	2.58	0~10	0.305^**^	1	
3. Optimism	12.92	2.45	5~24	0.296^**^	0.206^**^	1

** *P < 0.01*.

### Predictors of Life Satisfaction

**Figure 2:**
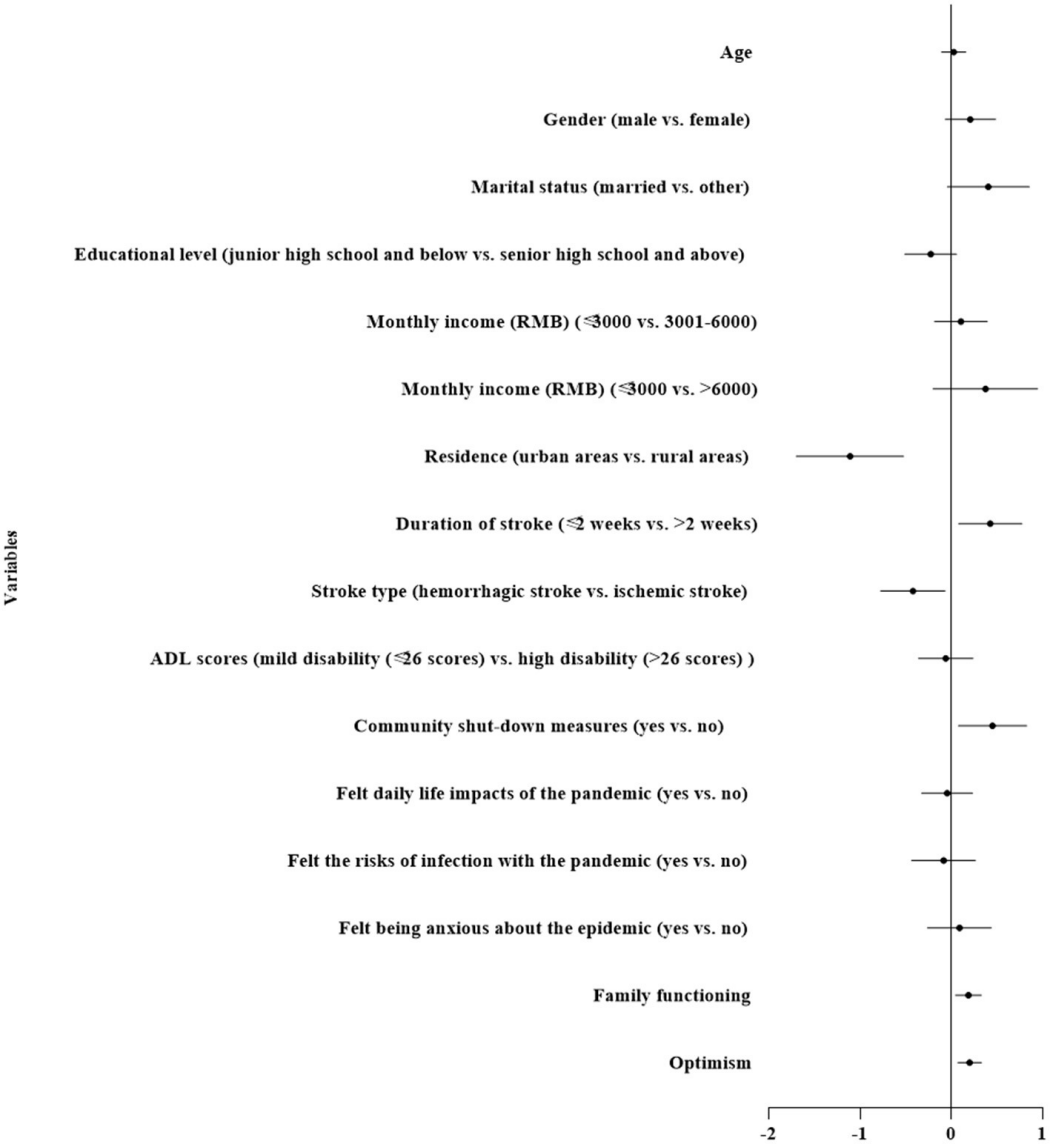
The forest plot of associate factors of life satisfaction.

Table 3, Figure 2 illustrates the final results of the HMR models of stroke patients' life satisfaction. A total of 25.1% of the variance was explained by the final model. Results from the *R*^2^ change indicated that the variance explained by each block of variables was 15.4, 3.0, 2.9, and 3.8% for demographic and clinical characteristics, pandemic responses, family functioning, and optimism, respectively. Living in the rural areas (β = −1.110, 95% CI−1.698-−0.522, *P* < 0.001) and ischemic stroke (β = −0.420, 95% CI−0.772-−0.069, *P* < 0.05) were observed to decrease life satisfaction, while more than 2 weeks duration of stroke (β =0.428, 95% CI 0.080–0.776, *P* < 0.05) and from an area without community shut-down measures (β = 0.452, 95% CI 0.077–0.827, *P* < 0.05) were observed to increase life satisfaction. Moreover, stroke patients who had better family functioning (β = 0.188, 95% CI 0.047–0.330, *P* < 0.01) and optimism (β = 0.202, 95% CI 0.073–0.331, *P* < 0.01) were observed to experience higher life satisfaction.

**Table 3:** The hierarchical regression analysis of life satisfaction (*N* = 207).

**Variables**	**Beta**	**Standardized beta**	**95%CI of beta**	***t-*value**	***P* value**	***R* ^2^**	**Δ*R^**2**^***
**Block1 demographic and clinical characteristics**						0.154	0.154
Age	0.026	0.026	-0.107 0.160	0.390	0.697		
Gender (Male vs. Female)	0.209	0.101	-0.066 0.485	1.498	0.136		
Marital status (Married vs. Other)	0.406	0.126	-0.042 0.855	1.785	0.076		
Educational level (Junior high school and below vs. Senior high school and above)	-0.225	−0.113	-0.508 0.058	−1.567	0.119		
Monthly income (RMB) (≤3,000 vs. 3,001–6,000)	0.106	0.053	-0.184 0.395	0.718	0.474		
MMmonthly income (RMB) (≤3,000 vs.3,001–6,000)	0.375	0.091	-0.198 0.948	1.291	0.198		
Residence (Urban areas vs. Rural areas)	-1.110	−0.260	-1.698 -0.522	−3.724	0.000^**^		
Duration of stroke (weeks) (≤2 weeks vs. >2 weeks)	0.428	0.182	0.080 0.776	2.424	0.016^*^		
Stroke type (Hemorrhagic stroke vs. Ischemic stroke)	-0.420	−0.169	-0.772 -0.069	−2.357	0.019^*^		
ADL scores (Mild disability (≤26 scores) vs. High disability (>26 scores)	-0.059	−0.030	-0.358 0.240	−0.391	0.696		
**Block2 epidemic responses**						0.184	0.030
Community shut-down measures (Yes vs. No)	0.452	0.184	0.077 0.827	2.375	0.019^*^		
Felt daily life impacts of the pandemic (Yes vs. No)	-0.045	−0.022	-0.325 0.235	−0.315	0.753		
Felt the risks of infection with the pandemic (Yes vs. No)	-0.084	−0.036	-0.434 0.266	−0.472	0.638		
Felt being anxious about the epidemic (Yes vs. No)	0.090	0.040	-0.261 0.441	0.505	0.614		
Block3 Family functioning	0.188	0.188	0.047 0.330	2.630	0.009^**^	0.213	0.029
Block4 Optimism	0.202	0.202	0.073 0.331	3.095	0.002^**^	0.251	0.038

* *P < 0.05*;

** *P < 0.01*.

## Discussion

### Principal Findings

Although the COVID-19 pandemic in China was nearly quelled after 3 months, a paucity of research has been conducted on the extent to which the pandemic affected stroke patient's subjective wellbeing in hospitals. More importantly, this survey represents the first cross-sectional study on the association of family functioning and optimism with life satisfaction among the population of stroke patients during the COVID-19 pandemic. The present study showed that the levels of life satisfaction in Chinese stroke patients was higher than those in general public of other countries and university students in Poland during the first peak of COVID-19 pandemic (34, 35). The reason for this phenomenon might be that COVID-19 outbreak was under control with serene and spacious medical environment (36). Shenyang was less influenced by COVID-19 pandemic and was in a low risk are in China compared with others (37). Besides, during the COVID-19 pandemic, a series of hospitals have provided prevention and management protocols to offer quality and continuous care for stroke patients, such as providing green channels with personal protective equipment, implementing group management in the process of diagnosis and treatment to avoid cross-infection (38), stroke patients with non-infectious were provided in separate rooms with more meticulous, assured health and psychological care, which resulted in the enhancement of life satisfaction (39).

The results from this study indicate that demographic and clinical factors were critical to interpret life satisfaction among stroke patients, accounting for 15.4% of the observed variance in stroke patient's subjective wellbeing. Furthermore, individual's internal support or psychological resources like family functioning and optimism, played critical roles in promoting life satisfaction, which corroborates previous research illustrating that positive beliefs could affect the appraisal of the stress response, help individuals facilitate and adapt to the stressful settings and tackle difficulties, which could improve stroke patient's life satisfaction (40).

Residence, duration of stroke, stroke type, and epidemic responses with community shut-down measures were strong predictors of life satisfaction for stroke patients during the COVID-19 pandemic. Stroke patients living in cities experienced slightly higher levels of life satisfaction during the COVID-19 pandemic. The most likely reason is that, Chinese stroke patients living in urban areas tend to obtain quality medical care in tertiary hospitals, which would be conducive to current life quality, psychological health, and subjective wellbeing (41). A longer stroke duration was positively associated with life satisfaction, which is in accordance with a previous research suggesting that the patients have adapted to the disease control and management (42). Ischemic stroke was a risk factor for poor life satisfaction among stroke patients. This may be because patients with ischemic stroke are more prone to hemiplegia, slurred speech, crooked mouth and other clinical symptoms, leading to a poorer prognosis and thus may cause ischemic stroke patients to have a lower subjective wellbeing (43). In addition, stroke patients who did not experience community shut-down measures during the COVID-19 epidemic were more satisfied with their life, which has been also shown by Fan et al. (44).

This study found that family functioning was moderately and positively associated with life satisfaction, which confirmed that a higher familial sense of togetherness, familiarity, and satisfaction with family ties contributed to improved psychological wellbeing for stroke patients when they faced a public health emergency (21, 45). Most research has shown that people who have stronger familial cohesion and communication have more positive perceived family roles and responsibilities, and thus, higher life satisfaction (46, 47). Conversely, poor family relationships and negative caregiving experiences could decrease the sense of life satisfaction for stroke patients. Accordingly, sufficient family support and positive experiences of caregiving from both family caregivers and hospital workers were related to higher life satisfaction for stroke patients during the COVID-19 epidemic.

In this study, optimism was positively associated with life satisfaction, which was in agreement with studies showing that optimistic stroke patients were more likely to have higher subjective wellbeing during the COVID-19 pandemic (48). The greater family support that stroke patients received, the higher optimism levels they had, which made stroke patients believe that positive events would continuously and universally take place, thereby increasing their life satisfaction (49). Attribution theory states that individuals with optimism appraise difficult circumstances (e.g., the COVID-19 pandemic) in a positive way and have a more hopeful outlook regarding the future, which leads to improved life satisfaction (50). Conversely, patients with low levels of optimism might tackle the difficult events in a more negative way and have bleak expectations for their future, which could result in the reduction of life satisfaction among stroke patients (51). Therefore, investment in enhancing optimism and family functioning may be effective in reducing the negative effects of public health emergencies and improving perceived subjective wellbeing.

## Limitations

There are several limitations in the present research. First, the data were selected from one hospital in Shenyang with a relative small sampling, which did not allow for generalization of the findings to stroke patients in other areas, or globally, during the COVID-19 pandemic. Therefore, expanding hospitals and participants to increase the results of generalizability should be prominent in the future study. Second, this was a cross-sectional study, thus, the causal relationships between optimism, family functioning, and life satisfaction require further research. Therefore, future research should utilize a longitudinal design during the pandemic to better establish the direction of relationships between the research variables.

## Conclusion

The life satisfaction of Chinese stroke patients was relatively high. Family functioning and optimism exerted strong positive association with life satisfaction. This study contributes to a better perspective on the protective factors of family functioning and optimism on life satisfaction among stroke patients during the COVID-19 pandemic. The training on the skills of family functioning and optimism enhancement intervention should be provided in order to elevate of life satisfaction for stroke patients.

Therefore, optimism intervention including face-to-face or online training conducted by training facilitators with series activities in-person and group-based should be conducted to improve stroke patients' levels of optimism, enhancing their ability to cope with stress adversities, improving positive psychological resources and establish a preventive mechanism without suffering mental illness and finally improve their life satisfaction (52). In addition, given the influence of family function on life satisfaction among stroke patients, it is necessary to customize family service programs to improve effective connections and positive interaction within the family, giving patients more spiritual and material support and reducing the burden of family care, thereby improving the family relationship and function, increasing patient's life satisfaction and promoting superior recovery.

## Data Availability Statement

The datasets generated for this study are available on request to the corresponding author.

## Ethics Statement

The studies involving human participants were reviewed and approved by China Medical University. The patients/participants provided their written informed consent to participate in this study.

## Author Contributions

YS and CC contributed to acquisition and analysis of data, drafting, and revision of the manuscript. YJ contributed to revision of the manuscript. WZ contributed to acquisition and analysis of data. LM contributed to acquisition data and the statistical analysis. KS contributed to revision of the manuscript and provide English edits. YX contributed to acquisition of data. XY was responsible for the conception and design. All authors contributed to the article and approved the submitted version.

## Funding

This work was supported by the fellowship of China Postdoctoral Science Foundation [Grant #2020M681022] and Liaoning Provincial Education Office of China, Serving Local Projects [FWRW2020004].

## Conflict of Interest

The authors declare that the research was conducted in the absence of any commercial or financial relationships that could be construed as a potential conflict of interest.

## Publisher's Note

All claims expressed in this article are solely those of the authors and do not necessarily represent those of their affiliated organizations, or those of the publisher, the editors and the reviewers. Any product that may be evaluated in this article, or claim that may be made by its manufacturer, is not guaranteed or endorsed by the publisher.

## Abbreviations

COVID-19: The Coronavirus Disease-19
SWLS: Satisfaction with Life Scale
LOT-R: Revised Life Orientation Test
APGAR: Family Adaptation, Partnership, Growth, Affection, and Resolve
ADL: Activities of Daily Living
HMR: Hierarchical Multiple Regression.
